# Barriers and Facilitators in the Implementation of a Syndromic Antibiogram for Pediatric Patients Hospitalized in Maputo, Mozambique: A Qualitative Study Using the Dynamic Adaptation Process (DAP) Framework

**DOI:** 10.3390/antibiotics15020178

**Published:** 2026-02-06

**Authors:** Darlenne B. Kenga, Troy D. Moon, Mohsin Sidat, Valéria Chicamba, Andrea Ntanga Kenga, Yara Manjate, Dércio Nhanala, Inês C. Caetano, Ramígio Pololo, Olga Cambaco, Jahit Sacarlal

**Affiliations:** 1 Department of Microbiology, Faculty of Medicine, University Eduardo Mondlane, Maputo 257, Mozambique; yaramanjate.ym@gmail.com (Y.M.); nhanaladercio26@gmail.com (D.N.); inesdacaetano@gmail.com (I.C.C.); jahityash2002@gmail.com (J.S.); 2 Department of Tropical Medicine and Infectious Diseases, Tulane University Celia Scott Weatherhead School of Public Health and Tropical Medicine, New Orleans, LA 70112, USA; tmoon2@tulane.edu (T.D.M.); cambacoolga@gmail.com (O.C.); 3 Department of Community Health, Faculty of Medicine, University Eduardo Mondlane, Maputo 257, Mozambique; mmsidat@gmail.com; 4 Pediatric Intensive Care Unit, Hospital Central de Maputo, Maputo 1100, Mozambique; vickyy.valeria@gmail.com; 5 National Institute of Health, Marracuene 493, Mozambique; andrea.kenga@ins.gov.mz (A.N.K.); ramigio.pololo@ins.gov.mz (R.P.)

**Keywords:** barries, facilitators, syndromic antibiogram, pediatric, dynamic adaptation process, Mozambique

## Abstract

**Introduction:** The global rise in antimicrobial resistance poses a growing threat to public health, particularly in low- and middle-income countries where diagnostic capacity and surveillance systems remain limited. In these settings, optimizing empiric antibiotic prescribing is critical, and syndromic antibiograms offer a promising approach to support evidence-based decision-making. This study examines anticipated barriers and facilitators to the adoption of syndromic antibiograms from the perspectives of pediatric clinicians and laboratory professionals at Maputo Central Hospital in Mozambique. **Methods:** Guided by the Dynamic Adaptation Process (DAP) framework, this qualitative study used semi-structured interviews with eighteen healthcare professionals to explore empiric antibiotic prescribing practices, perceptions of syndromic antibiograms, and system-level barriers and facilitators. Data were analyzed thematically using deductive codes derived from the DAP framework alongside inductive codes generated from participants’ narratives. **Results:** Barriers were identified at individual, organizational, and systems levels. Individual barriers included limited awareness, reliance on traditional practices, and resistance to change. Organizational barriers included weak leadership support, insufficient training, poor communication between clinicians and laboratory staff, suboptimal sample collection, heavy workloads, and staff shortages. Systems-level barriers comprised shortages of laboratory supplies and medicines, delays in laboratory results, and weak monitoring mechanisms. Facilitators included health worker motivation for evidence-based practice, organizational collaboration, peer and team support, and the presence of influential champions. Systems-level enablers included functional laboratory services, supportive institutional environments, alignment with clinical guidelines, and recognition of clinical utility. **Conclusions:** Successful implementation of syndromic antibiograms in LMIC will require addressing systemic and organizational barriers while fostering professional motivation, collaboration, and institutional support. Sustainable integration will depend on coordinated strategies—including resource strengthening, continuous training, supportive leadership, and structured monitoring—that collectively strengthen antimicrobial stewardship and inform health policy.

## 1. Introduction

The global rise in antimicrobial resistance (AMR) represents a critical public health challenge, particularly in low- and middle-income countries (LMICs), where diagnostic capacity and antimicrobial surveillance systems are often underdeveloped [1,2]. Rational antibiotic use is especially vital in pediatric populations, who are more susceptible to both infections and the adverse effects of inappropriate treatment [3,4].

A Cochrane systematic review confirmed that interventions designed to reduce inappropriate antibiotic use among hospitalized patients can significantly reduce AMR while improving microbiological and clinical outcomes [5]. However, most existing studies originate in high-income countries, leaving a substantial gap in the evidence on the effectiveness and key components of antimicrobial stewardship programs (ASP) in LMICs [6].

In this context, syndromic antibiogram tools, which compile local antimicrobial susceptibility data by clinical syndrome rather than by specific pathogen, have emerged as a promising approach to guide empiric antibiotic therapy in settings where timely culture results are rarely available. Ensuring accurate and consistent data entry into platforms such as WHONET is critical for this process, as these systems aggregate and analyze microbiological data in standardized formats, enabling the reliable generation of resistance patterns that underpin the development and maintenance of syndromic antibiograms [7,8].

Moreover, limited attention has been paid to the social and contextual factors that shape the implementation of syndromic antibiograms in resource-constrained settings. These factors encompass barriers and facilitators operating across health systems, clinical workflows, and community contexts. Understanding these implementation dynamics is particularly important in pediatric services within LMICs, where the burden of infectious diseases remains high, and laboratory infrastructure capacity is often insufficient [9]. In Mozambique, [*Hospital Central de Maputo* (HCM)], the country’s largest quaternary referral center, relies heavily on empiric antibiotic prescribing, largely due to delays in microbiological results and underutilized diagnostic resources [10,11,12]. In such settings, syndromic antibiograms may provide a practical, context-specific approach to guide more appropriate antimicrobial therapy by linking local susceptibility patterns with common clinical presentations [7,13,14]. However, their implementation remains challenging due to fragmented data systems, limited clinician awareness of syndromic antibiogram use and interpretation, weak coordination between clinical and laboratory teams, and gaps in antimicrobial stewardship infrastructure. Despite these barriers, factors such as strong institutional leadership, targeted training initiatives, and alignment with broader stewardship strategies can facilitate successful adoption [2,15,16].

This study aims to identify the key factors influencing the use of the syndromic antibiogram in managing hospitalized children with suspected bacterial infections. The findings will help inform more effective implementation strategies and support evidence-based antimicrobial practices in LMIC settings.

## 2. Results

The findings presented in this section reflect participants’ anticipated and perceived barriers and facilitators to the implementation of the syndromic antibiogram, based on their experiences, expectations, and assessments of feasibility prior to implementation.

A total of 18 participants were included in the study, comprising healthcare professionals from different disciplines within the pediatric care setting. Of these, 10 were physicians, including one who served as the care director of the pediatric department. Five were nurses, and three were laboratory technicians. The participants were predominantly female (72.2%), and all had more than six years of experience in their jobs (Table 1).

### 2.1. Mapping of Barriers and Facilitators According to the DAP Framework Phases

**Figure 1:**
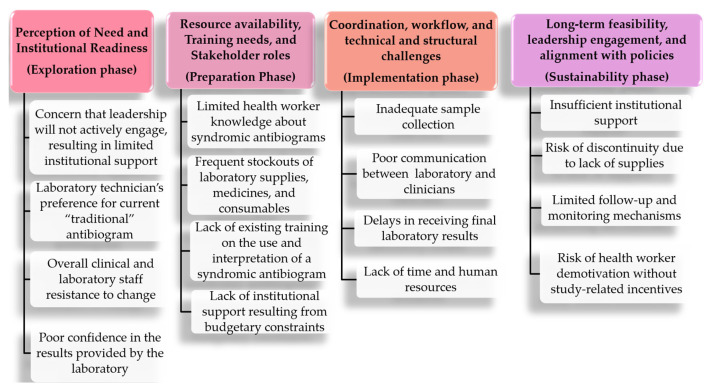
Mapping of perceived barriers by themes, using the DAP framework.

**Figure 2:**
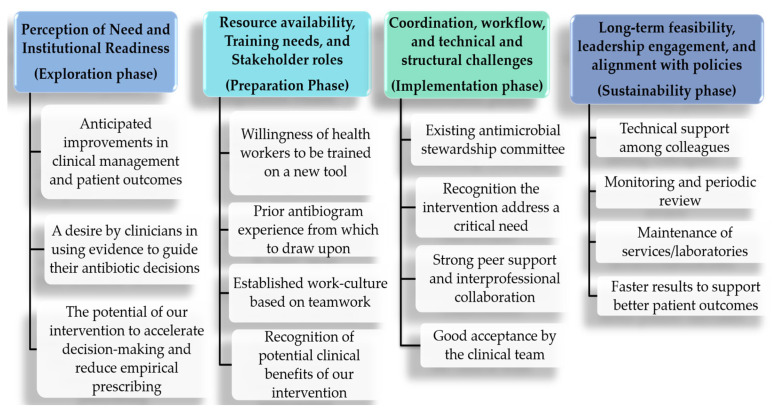
Mapping of perceived facilitators by themes, using the DAP framework.

To guide interpretation of our findings, we employed a hybrid deductive–inductive thematic approach, allowing themes to emerge from the data through systematic coding of semi-structured interviews rather than being imposed a priori. Transcripts were coded to identify anticipated and perceived barriers and facilitators to implementing the syndromic antibiogram. Once developed, these data-driven themes were mapped onto the four sequential phases of the Dynamic Adaptation Process (DAP) framework to support structured interpretation (Figure 1 and Figure 2), illustrating how barriers and facilitators aligned with each phase. Table 2 summarizes the most salient barriers and facilitators identified for each phase. This approach allowed the DAP framework to serve as an organizing lens rather than a prescriptive structure, providing a holistic understanding of the contextual factors shaping adaptation within the hospital environment.

#### 2.1.1. Exploration Phase-Perceptions of Need and Institutional Readiness

The following results summarize the main barriers and facilitators to implementing the syndromic antibiogram, as reported by physicians, nurses, and laboratory technicians prior to its introduction (Figure 1 and Figure 2).

Participants identified four principal barriers that, if not adequately addressed, could impede the implementation of the syndromic antibiogram intervention: (1) limited institutional support; (2) laboratory staff’s reluctance to modify their established routine; (3) broader resistance to change among clinical and laboratory teams; and (4) lack of confidence in the laboratory results provided by the laboratory.

Participants expressed concerns that hospital leadership may not take an active role in promoting or coordinating the implementation of the syndromic antibiogram. Such limited engagement could weaken institutional support, hinder early planning, and reduce motivation among both clinical and laboratory teams.

*“A long time ago, there was an opportunity to do antibiograms, but in recent years, due to a shortage of resources, it is no longer done. For some time now, we have been unable to request blood cultures, and as a result, people have lost confidence in the results. If not coordinated from the top, there will always be difficulties.”*

(Physician 4)

Furthermore, laboratory technicians demonstrated initial resistance to adopting the new approach, expressing a preference for the “traditional” antibiogram and discomfort with changing established routines.

*“I would rather continue with the traditional method. The new approach will bring an excessive workload, especially in the first months or even years of implementation.”*

(Laboratory Technician 1)

One health worker noted that transitioning to new systems requires time and adjustment, explaining that uncertainty around unfamiliar processes can undermine confidence and reduce staff willingness to engage with innovations. This insight highlights the broader challenge of implementing new interventions in settings where professionals are more comfortable with established routines.

*“Some people are still attached to old routines and are reluctant to change. It takes time for professionals to adapt to new tools and ways of working. Change often creates uncertainty, and not everyone feels confident embracing new practices.”*

(Physician 6)

Some participants expressed low confidence in the laboratory system, citing limited capacity and inconsistent test performance as undermining trust in microbiology results. As one physician noted:

*“We would need to convince colleagues that it [the syndromic antibiogram] was developed with good quality. Many of the existing gaps are linked to the limited microbiological capacity of the main hospital, which affects the credibility of laboratory results and confidence among clinicians.”*

(Physician 1)

Participants consistently emphasized the importance of strengthening laboratory capacity and rebuilding trust prior to implementation. These concerns were perceived to persist due to historical underinvestment in laboratory services and limited opportunities for sustained interaction between clinicians and laboratory personnel, which have shaped skepticism toward laboratory-generated data.

*“To ensure the success of the syndromic antibiogram, we must first build trust in the quality of its development and strengthen the hospital’s microbiological capacity. Without these foundations, it will be difficult to encourage clinicians to rely on and use its findings in practice.”*

(Physician 3)

Moreover, healthcare workers identified three key facilitators that could support the implementation of the syndromic antibiogram tool, including: (1) anticipated improvements in clinical management and patient outcomes as a result of its use; (2) a desire by clinicians in using evidence to guide their antibiotic decisions; and (3) the potential of our intervention to accelerate clinical decision-making and reduce empiric prescribing.

*“This tool would be extremely useful, as it would reduce empiric antibiotic prescribing, which is currently based primarily on the patient’s clinical presentation and the suspected pathogen. For example, in cardiology, common pathogens in endocarditis range from Streptococcus to Staphylococcus, and sometimes fungi, with treatment choices often adjusted according to the patient’s response (such as persistent fever or the need to escalate therapy). Having this instrument would allow us to decrease empiric prescribing and avoid inappropriate antibiotic use, thereby promoting a more precise and efficient therapeutic approach.”*

(Physician 2)

The participants underscore that, although current clinical practice already relies on syndromic management, the introduction of a syndromic antibiogram would enhance the rationality of antibiotic prescribing. By providing locally informed evidence, the tool is perceived as a means to shift decision-making from reliance on clinical judgment alone toward a more evidence-based approach.

*“What we do now is essentially syndromic treatment, but the syndromic antibiogram would help rationalize medication use. It will guide us toward more evidence-based prescribing rather than relying solely on clinical judgment.”*

(Physician 8)

Health workers also highlighted the potential of syndromic antibiograms to strengthen clinical decision-making. The tool was seen as a means to make antibiotic prescribing more systematic, reduce reliance on empiric judgment, and promote the use of microbiological evidence, thereby improving alignment between prescribed therapies and patients’ actual treatment needs.

*“I think it will help; it is very important to have this instrument here in our sector. It will make antibiotic prescribing more systematic and encourage testing to identify the most appropriate treatment for each patient.”*

(Physician 5)

#### 2.1.2. Preparation Phase-Resource Availability, Training Needs, and Stakeholder Roles

During the preparation phase, physicians, nurses, and laboratory technicians identified several potential barriers and facilitators that could affect the successful implementation of the syndromic antibiogram, including: (1) limited health worker knowledge about syndromic antibiograms; (2) frequent stockouts of laboratory supplies, medicines, and consumables; (3) lack of specific training for clinicians on the use and interpretation of the syndromic antibiogram; and (4) uncertain leadership engagement. Participants noted that limited understanding of syndromic antibiograms and their potential use constrained their ability to fully engage with the process.

*“I don’t have knowledge regarding this antibiogram; I will need training. Honestly, I have never heard of a syndromic antibiogram before, so I would need more information and guidance to understand how it works.”*

(Physician 10)

Frequent shortages of laboratory supplies, including reagents, medicines, and essential consumables, were also frequently mentioned as potential constraints to implementing the syndromic antibiogram. Participants emphasized that even when clinical teams were motivated to utilize laboratory data, the lack of consistent microbiological testing capacity limited their ability to obtain reliable results. As one laboratory technician explained:

*“Many of the challenges we face are linked to the hospital’s limited microbiological capacity. For instance, we have spent almost a year without reagents for many culture tests, and sometimes we cannot perform them at all because there are no reagents or even medicines available.”*

(Laboratory Technician 2)

Some participants expressed concerns about the quantity of clinical samples that would be used to generate the syndromic antibiogram. They noted inconsistent sample-collection practices and raised questions from the group of health workers interviewed.

*“I don’t know how many patients will manage to have samples collected for the different syndromic presentations. The challenge is that sample collection often depends on the availability of staff and materials, which are not always consistent. Sometimes, even when patients are eligible, the lack of resources prevents proper sample collection.”*

(Physician 7)

One participant described a persistent belief that the hospital lacks adequate institutional support, a challenge attributed to chronic budget limitations and a dependence on external donations or partner-driven initiatives. This constrained environment fosters doubt among clinical staff about what can realistically be achieved with existing resources. Despite ongoing activities and years of experience, participants felt that institutional progress had largely stagnated, reinforcing the perception that expectations continue to exceed available support.

*“There is a perception that institutional support is insufficient, often due to budgetary constraints and a reliance on donations or external assistance. The prevailing sentiment is that limited action is taken because resources are scarce, which raises questions about what more could realistically be achieved, particularly when local practices are compared with externally funded initiatives, collaborations, or competitive programs. Overall, the impression remains that, despite time and accumulated experience, the institutional response continues to fall short of what is expected.”*

(Physician 3)

Several facilitators were also identified, including: (1) the willingness of participants to be trained; (2) prior experience with antibiograms and recognition of the tool’s importance; (3) the importance of working together as a team; and (4) acknowledgment of the clinical benefits the antibiogram could bring. Participants emphasized that additional training would be a crucial step to strengthen their knowledge and skills, and to support them in using the syndromic antibiogram tools.

*“We are available, and although training is undeniably necessary and a brief orientation accompanies each introduction, the response from human resources to this training is expected to be favorable in terms of attendance and dedication, because we are committed to this effort.”*

(Care Director)

Previous exposure to other types of antibiograms was described as a useful background experience for engaging with the syndromic approach. Additionally, many recognized the relevance of the syndromic antibiogram to improving pediatric care, particularly in contexts where empirical prescribing is common, and its potential clinical benefits were also frequently reported. One physician reflected on a prior experience abroad:

*“This was part of the work I had in the United States. Each hospital maintains its own antibiogram, thereby monitoring the prevalence of MRSA among isolated Staphylococcus aureus strains. They track resistance frequencies among Gram-negative bacteria. Subsequently, they assess the specific patterns of resistance and the susceptibility to antibiotics. Thus, you have a local antibiogram that informs local clinical practices. It can vary from state to state and from city to city.”*

(Physician 4)

Teamwork was highlighted as a key factor supporting collective engagement during the preparatory stage. Participants attributed this to established collegial relationships and shared professional responsibility, which enabled coordinated planning and mutual support even in the absence of formal implementation structures.

*“We conduct daily medical ward rounds, during which all team members are encouraged to ask questions, raise concerns, offer corrections when necessary, and contribute their opinions respectfully. After the ward round, we continue discussions in smaller groups, reviewing patient cases, consulting the literature, and clarifying uncertainties. This practice is actively encouraged and taught, as we are a teaching hospital. Younger clinicians are guided to ask questions and seek support whenever they encounter limitations, as the lives of patients depend on informed and collaborative decision-making.”*

(Care Director)

One participant emphasized that improved diagnostic guidance, such as consistent access to culture and susceptibility data, could meaningfully strengthen clinical decision-making in the department. They underscored that greater precision in antibiotic selection may help prevent severe outcomes and support clearer communication with families, highlighting a strong belief in the tool’s potential to enhance the quality of pediatric care.

*“This tool would be well received in our department, as adverse outcomes are a recurrent concern under current conditions. If we had the capacity to perform cultures and antibiograms consistently, clinicians would be able to adjust treatments with greater precision, potentially saving patients’ lives and more effectively involving families in the decision-making process.”*

(Care Director)

#### 2.1.3. Implementation Phase-Coordination Processes, Workflow Integration, and Technical and Structural Challenges

Throughout the implementation phase, physicians, nurses, and laboratory technicians identified several key barriers that could hinder the integration of the syndromic antibiogram into routine practice. For instance, participants described resistance among colleagues as a challenge to adopting new approaches. Additional barriers included difficulties in coordinating workflows, integrating the syndromic antibiogram into existing processes, and addressing technical and structural limitations within the hospital setting.

*“Anything new is, at times, not viewed very favorably; people tend to become complacent with existing difficulties, or they conclude that ‘this may not significantly improve our practical outcomes,’ or it may be perceived as a waste of time, or as something that ‘is attractive in theory but cannot be implemented effectively in practice.”*

(Physician 2)

Participants also highlighted several practical challenges related to specimen collection, storage, and transport that could affect the successful implementation of the syndromic antibiogram. They noted that sample integrity is often compromised by issues such as suboptimal storage conditions, inconsistent processing times, and limited communication between clinical teams and the laboratory. Both clinicians and nurses emphasized that improved coordination, along with reliable, appropriate storage solutions on the wards, would be critical for the syndromic antibiogram intervention to work.

*“Nurses mainly collect samples, but medication timing can affect accuracy. Samples may need special storage, and current refrigerators might not be suitable. Quick processing is crucial, and more specific storage solutions might be needed depending on the sample type.”*

(Physician 3)

*“Perhaps coordinating with the laboratory to establish better coordination and communication would be beneficial. If there’s a ward that, for example, has a small freezer or a small cooler where we can store all the labeled samples within 24 h so that, for example, the next day or at a designated time X, they can be processed, that would help a lot.”*

(Nurse 2)

Participants further anticipated that weak communication channels between laboratory staff and clinicians could become a barrier during implementation, potentially negatively impacting the flow of information that is needed for clinical decision-making once the syndromic antibiogram is introduced.

*“When clinicians request tests, they’re never available. I requested the test, and it still isn’t available. Then I sent the sample, but I never got the result, so I gave up requesting it. I adjust my work mentally, assuming I will never receive the result for that test. Later, the laboratory may improve and make the test available, but it will face a challenge, because the person [clinician] has already lost the habit of requesting that test, since it was never available, and they never received results.”*

(Physician 8)

Limited time and insufficient human resources were also identified as obstacles to effective implementation. Participants noted that staffing constraints often delayed or prevented timely sample collection, potentially undermining the ability to guide treatment decisions. These challenges were perceived to persist due to longstanding workforce shortages and high clinical workloads, which constrain adherence to optimal diagnostic timelines in routine practice.

*“Delaying the initiation of antibiotic therapy in order to ensure optimal specimen collection represents a major challenge, particularly due to insufficient staffing to collect samples on time, that is, before or at least within 24 h of starting antibiotic treatment.”*

(Physician 7)

Additionally, the participant underscores a persistent shortage of personnel, highlighting how current staffing constraints hinder the efficiency of laboratory workflows. At present, data are manually extracted from the laboratory register by a single technician, a process that is time-consuming, labor-intensive, and prone to delays or inaccuracies. The participant notes that assigning a dedicated staff member to enter data directly into the WHONET platform would significantly increase efficiency and streamline operations. This perspective reinforces the essential role of adequate human resources and specialized data-management support in ensuring the timely and reliable generation of microbiological data, which is crucial for the effective implementation of tools such as the syndromic antibiogram.

These challenges were perceived to persist in the context of chronic understaffing and the lack of institutionalized roles for laboratory data management, which constrain the routine use of digital platforms such as WHONET.

*“We have also experienced a shortage of personnel. Currently, the laboratory technician retrieves data manually from the laboratory register. However, having a dedicated staff member specifically assigned to enter the data directly into WHONET platform would make the process much more efficient and greatly improve our workflow.”*

(Laboratory technician 1)

Several facilitators were identified, including (1) an existing antibiotic stewardship committee, (2) recognition that the intervention could address a critical need within pediatrics, (3) peer support and interprofessional collaboration, and (4) clinical team acceptance. Participants felt these were sustained by prior stewardship experience and established working relationships, which fostered trust, shared ownership, and openness to locally developed decision-support tools.

*“The hospital already has a committee for the rational use of antibiotics, so there may be influential people who can help implement this initiative and act as champions for it. Their involvement would make it easier to gain acceptance and support from other staff members.”*

(Physician 5)

Participants recognized that the syndromic antibiogram addresses a critical clinical need in the pediatrics department and noted high staff interest and anticipated receptivity, indicating strong pre-implementation acceptability. They also highlighted existing educational and collaborative structures, such as ongoing training cycles and weekly multidisciplinary clinical sessions, that provide natural platforms for integrating new tools and practices.

*“Yes, the antibiogram is a primary need in this department, and we are interested in having it implemented. There is already a positive indication, as the level of receptivity is expected to be high. Moreover, implementation is feasible since we already have continuous training cycles and weekly clinical sessions for both physicians and nurses of different categories. Therefore, there are already existing platforms, through regular trainings, work sessions, and lectures, where such initiatives can be discussed and integrated.”*

(Care Director)

In addition, peer support was highlighted as a mechanism that strengthened collective participation. Participants perceived this support to be sustained by established collegial relationships and a shared sense of accountability within clinical teams, particularly in contexts where formal institutional support is limited.

*“During clinical rounds, we discuss each case as a team, assess the patient’s condition, and reflect on possible interventions to improve their clinical outcomes. If the patient does not show satisfactory progress, we conduct additional studies and revisit the case discussion continuously and collaboratively.”*

(Physician 6)

*“We have a dedicated research unit responsible for the coordination and implementation of all research activities within the department. Even when a study does not originate directly from the unit, it must still be coordinated if it holds relevance or potential benefit for the department. The research unit provides valuable support through a multidisciplinary team composed of physicians and nurses who contribute as needed to facilitate the effective conduct of research activities.”*

(Care Director)

Participants also recommended that (1) placing physical copies of the syndromic antibiogram in visually accessible locations, (2) introducing a system for acknowledgment of the staff with the top performance/use of peer influence, and (3) designating specific personnel who would be responsible for data entry in the WHONET platform could further help in the successful implementation of the intervention.

*“I would say that this tool should be presented in the form of a leaflet or poster that we can display in our offices and treatment rooms, serving as a reminder to help us monitor and recognize when we are exceeding the recommended duration of antibiotic treatment.”*

(Nurse 3)

The participant indicates that the syndromic antibiogram is likely to be positively received by the majority of clinicians, suggesting strong initial acceptability within the institution. This openness creates a favorable environment for implementation. They also highlight the presence of potential “local champions”, influential individuals who can advocate for the initiative and help mobilize colleagues. The mention of an existing committee dedicated to the rational use of antibiotics further suggests institutional structures that could support and legitimize implementation efforts. Together, these elements signal a supportive organizational climate in which motivated stakeholders and established governance bodies could play critical roles in promoting the successful adoption and integration of the tool.

*“Most clinicians in the hospital would well receive a tool of this kind. In addition, there may be local champions who can support the initiative; the hospital already has a committee dedicated to the rational use of antibiotics, and individuals with influence within this context can serve as advocates for implementation.”*

(Physician 10)

#### 2.1.4. Sustainability Phase–Long-Term Feasibility, Leadership Engagement, and Alignment with Hospital Policies

Finally, participants identified several barriers which we mapped to the Sustainability phase: (1) insufficient institutional support, (2) risk of discontinuity due to lack of supplies, (3) limited follow-up and monitoring mechanisms, and (4) risk of staff demotivation if study incentives were not maintained over time.

Participants emphasized that weak institutional commitment, particularly in relation to procurement and supply management, would likely undermine continuity of the intervention over time. These barriers were perceived to result from fragile procurement systems and reliance on externally supported supply chains, which limit the hospital’s capacity to sustain routine laboratory operations beyond the duration of externally funded projects.

*“I’m not sure if it [the intervention] will be feasible, because the consumable materials we’ve been receiving lately are very limited… There’s always a shortage of everything. We don’t have consistent institutional support for these materials. Today, we might have one type of [culture] medium, but it could take another six months to get more. I think the institutional support system doesn’t really work, once supplies run out, that’s it.”*

(Laboratory technician 3)

One participant expressed strong support for the anticipated benefits of the syndromic antibiogram, acknowledging its potential value for both clinicians and patients. However, this optimism is tempered by a persistent concern regarding the lack of continuity that has characterized previous diagnostic or quality-improvement initiatives within the hospital.

*“We believe this initiative will be advantageous and beneficial for all of us. However, our main concern is that it may be introduced without any follow-up or continuity. What often happens here is the recurrent lack of reagents and essential materials. We start a project, an activity, or a diagnostic process, but then it stops midway due to the absence of consistent resources and sustainability.”*

(Care Director)

Limited follow-up and weak monitoring mechanisms were also described as constraints to sustaining progress. Participants clarified that monitoring was understood to encompass regular supervisory practices, including peer review of prescribing behavior, periodic review of syndromic antibiogram use, and informal performance checks to ensure adherence to agreed-upon clinical and laboratory procedures. In the absence of such oversight, accountability was perceived to diminish, particularly when responsibility for follow-up was not formally assigned.

These challenges were perceived to persist due to the lack of institutionalized monitoring structures, such as routine audits or feedback mechanisms, and competing clinical demands that limit sustained oversight.

*“Yes, there should be some form of monitoring among colleagues, because at times we tend to become less attentive when no one is supervising our work. However, when there is someone present to review and ensure that activities are progressing as expected, it can be very helpful. I believe such monitoring could significantly improve our performance and the proper use of this tool, ultimately leading to meaningful gains.”*

(Nurse 4)

Participants linked potential staff demotivation to the removal of study-related incentives after project completion. They noted that, in under-resourced settings, incentives have historically compensated for additional workload, making sustained motivation challenging once external support ends.

*“It will have an impact, but it is essential to engage in discussions with colleagues and help them understand that this is the way it should be, even though it might represent additional work. Incentives play an important role; they motivate and encourage commitment among staff. When people receive incentives, they tend to feel more motivated and engaged in their work.”*

(Laboratory technician 2)

One facilitator mapped to the Sustainability phase was existing opportunities for team-based patient or case discussions. Such peer support was seen as enhancing professional confidence and promoting continuity in using the syndromic antibiogram.

*“We conduct daily medical ward rounds, during which everyone has the opportunity to ask questions, provide feedback, and make corrections as necessary, all while maintaining professionalism and respect. After the ward round, we return to our respective areas to discuss the patients further, conduct research, and clarify any doubts. This is an activity that we actively encourage and use as a teaching moment, especially for the younger staff, as this is a teaching hospital.”*

(Care Director)

Participants also identified anticipated benefits of implementing the syndromic antibiogram that could support its long-term success, including enhanced confidence in clinical decision-making and earlier initiation of appropriate treatment, leading to improved patient outcomes. These expectations were consistently linked to prior positive experiences with institutionally endorsed antibiograms, which had historically provided trusted guidance and promoted consistency in prescribing practices.

*“The antibiogram used to come out annually, and I gained trust by following those institutional recommendations. It provided a sense of guidance and consistency in clinical decision-making. Having such reliable updates helped ensure that antibiotic prescriptions were aligned with current resistance patterns.”*

(Physician 1)

*“With the syndromic antibiogram, we will stop prescribing antibiotics empirically. Instead of rushing to broad-spectrum antibiotics, we will start with narrow-spectrum options supported by laboratory evidence.”*

(Physician 9)

*“It could help us manage infections more promptly, as we would not need to start a treatment that may ultimately be ineffective due to resistance. Instead of the patient’s condition deteriorating while waiting, we would be able to initiate the appropriate therapy immediately, based on the results. Therefore, I believe this approach would indeed be beneficial.”*

(Physician 7)

#### 2.1.5. A Concise Summary of the Most Salient Anticipated Barriers and Facilitators Identified for Each Phase

Table 2 synthesizes the most salient anticipated barriers and facilitators associated with each phase of the DAP framework for the implementation of a syndromic antibiogram. The table provides a concise overview of how the barriers and facilitators vary across the DAP phases, highlighting phase-specific considerations that are essential for planning, implementation, and sustainability of the syndromic antibiogram intervention.

**Table 2:** Most salient barriers and facilitators across phases of the Dynamic Adaptation Process (DAP).

DAP Phase	Key Barriers	Key Facilitators
Exploration	Limited knowledge of the syndromic antibiogram concept; lack of consolidated local data; low initial clinician awareness	Recognition of high antimicrobial resistance levels; perceived need to improve empirical prescribing; institutional interest in stewardship strategies
Preparation	Limited laboratory infrastructure; shortage of specialized human resources; absence of standardized protocols	Prior laboratory experience with conventional antibiograms; technical leadership support
Implementation	High workload; insufficient communication between laboratory and clinical services; challenges in integrating results into clinical practice	Engagement of key professionals; continuous training and capacity building; positive clinical feedback on the usefulness of the antibiogram
Sustainment	Dependence on external financial resources; staff turnover; lack of formalized institutional policies	Increasing institutional commitment; potential integration into stewardship programs; perceived positive impact on clinical decision-making

* *Note: The barriers and facilitators presented reflect anticipated factors identified prior to implementation*.

## 3. Discussion

This study explored anticipated barriers and facilitators to the implementation of a syndromic antibiogram within the pediatric inpatient services of HCM in Maputo, Mozambique. Importantly, the findings reflect institutional readiness, stakeholder expectations, and perceived feasibility prior to implementation, rather than post-implementation effectiveness. Using semi-structured interviews with 18 healthcare professionals, this study provides an in-depth understanding of the contextual, organizational, and behavioral dynamics that influence the adoption of this antimicrobial stewardship tool in a resource-limited setting. To the best of the authors’ knowledge, this is the first qualitative study conducted in Mozambique to examine the barriers and facilitators to integrating a syndromic antibiogram into the routine practices of a pediatric healthcare service.

Our use of the DAP framework allowed us to examine how implementation processes evolve through iterative interaction between contextual barriers and adaptive strategies. The DAP framework highlights four interrelated phases, namely: (i) exploration, (ii) preparation, (iii) implementation, and (iv) sustainability, each influenced by multiple ecological levels [17]. Applying this model facilitated the identification of how leadership engagement, institutional readiness, and human resource capacity could shape the trajectory of syndromic antibiogram implementation. Importantly, the DAP framework supports evidence-based adoption to local contexts, rather than being directly transferred. This principle is particularly relevant in LMICs such as Mozambique, where resource constraints and institutional healthcare working cultures may differ from those in high-income settings. During the exploration phase, barriers were mainly associated with limited prior awareness and understanding of the syndromic antibiogram among participants.

Several participants acknowledged the need for targeted training to improve their ability to interpret and apply microbiological data from the syndromic antibiogram in their clinical decision-making around antibiotic choice and duration of use. Similar gaps in knowledge have been reported in studies conducted in Tanzania, Kenya, Sri Lanka, South Africa, and Ghana, where insufficient training and weak integration of laboratory data into clinical practice have hindered antimicrobial stewardship efforts [16,18,19]. These findings highlight the importance of investing in continuous professional development programs focused on interpreting and using local microbiological evidence. Without such initiatives, the implementation of a syndromic antibiogram may remain superficial, failing to achieve its intended impact on clinical outcomes.

In the preparation phase, perceived barriers shifted towards limited leadership engagement. When leaders do not actively champion or coordinate an initiative, institutional commitment, planning, and resource allocation may be compromised [20,21,22]. Sustained support from hospital management and departmental heads is therefore crucial for motivating staff, reinforcing accountability, and facilitating collaboration across clinical and laboratory teams. While prior studies have shown that strong leadership commitment and role modeling are critical for sustaining ASP [20,22] the present study revealed a notable gap in leadership involvement and advocacy.

Our findings align with studies from Nigeria [23] and Saudi Arabia [24], which similarly highlighted insufficient managerial support as a key barrier to effective and sustainable antimicrobial stewardship programs. In resource-limited settings, leaders can act as change agents by prioritizing stewardship in institutional policies, mobilizing resources, and fostering a culture of continuous improvement. The DAP framework underscores this role, positioning leadership as a cross-cutting determinant across all stages of adaptation, capable of linking policy directives with operational realities and enhancing program sustainability.

Barriers anticipated during the implementation phase centered on interprofessional coordination and the absence of standardized communication and feedback mechanisms. Participants reported delays in feedback, inconsistent data-sharing mechanisms, and the absence of standardized reporting formats. Similar challenges have been reported elsewhere [25,26,27,28,29], with studies finding that weak communication channels between laboratory and clinical units limit the use of diagnostic data in guiding antibiotic therapy. This suggests that the successful implementation of the syndromic antibiogram in our context could depend not only on technological tools but also on strengthening organizational communication and interprofessional collaboration. Establishing systematic feedback loops and integrating electronic reporting systems could also enhance the timely flow of information and promote evidence-based decision-making [25,26,27].

Human resource limitations were also perceived as major constraints in the implementation phase of intervention success amongst our participants. They highlighted the lack of qualified personnel dedicated to data entry, result analysis, and, finally, the dissemination of the syndromic antibiogram amongst pediatric prescribers. This shortage reflects the broader systemic issue of understaffing in Mozambican hospitals, where clinical demands often outweigh surveillance and stewardship activities. Similar constraints have been observed in other sub-Saharan African contexts, where the scarcity of trained laboratory professionals compromised the continuity of quality improvement interventions [29,30]. Addressing this challenge requires long-term investment in workforce development, including the creation of specialized positions and mentorship structures to ensure the sustainability of stewardship initiatives.

When compared with previous studies in sub-Saharan Africa, our findings align with the well-documented persistence of systemic challenges, including weak laboratory–clinical integration, broad hospital resource constraints, and limited antibiotic stewardship infrastructure [21,30]. However, this study adds new insights by highlighting the adaptive behaviors and innovative practices employed by frontline professionals in our context. Our participants perceived that they could actively navigate through identified barriers through informal collaboration, improvisation, and a strong commitment to patient care. This demonstrated agency reinforces the value of participatory implementation strategies that leverage local expertise rather than relying solely on externally driven models of change.

Long-term sustainability was perceived as the greatest challenge to our intervention, with participants highlighting weak institutional support, donor-dependent supply chains, limited monitoring mechanisms, and concerns about staff demotivation following the withdrawal of study-related incentives. Similar sustainability challenges have been described in Nigeria and Saudi Arabia, where ASP struggled to persist once external funding diminished [23,24]. In our context, these barriers were perceived to persist due to fragile procurement systems, limited institutional ownership, and the absence of formalized accountability structures. Participants’ emphasis on monitoring as supervision, peer review, and routine performance feedback underscores the need for institutionalized oversight mechanisms to maintain long-term continuity.

Our findings also suggest that professional power dynamics may have influenced participants’ perspectives. Physicians tended to frame challenges in terms of clinical autonomy and urgency, whereas laboratory staff emphasized workload, resource constraints, and limited institutional recognition. Similar hierarchical dynamics have been described in the antimicrobial stewardship literature, where laboratory professionals often report feeling marginalized despite their critical role in generating microbiological evidence [15,31,32]. Such asymmetries may impact trust, communication, and the perceived legitimacy of laboratory-driven tools, underscoring the need for inclusive governance structures that elevate the voices of laboratories within stewardship initiatives.

Despite perceived structural and operational barriers, several facilitators were identified that we will leverage to support our intervention implementation. Participants highlighted strong teamwork and collegial relationships among physicians, pharmacists, and laboratory technicians at HCM, which were felt to facilitate problem-solving and mutual support in daily clinical routines. However, collaboration tended to occur primarily within, rather than across, disciplines. While this intra-professional cohesion supported workflow efficiency and enabled the teams to manage immediate challenges, it simultaneously revealed the limited collaboration and integration between clinical and laboratory staff. These findings are in contrast to other studies, which have emphasized that interprofessional collaboration across disciplines fosters trust, enhances adherence to stewardship recommendations, and promotes the optimal use of microbiological data [33,34,35].

Our study also found that intrinsic motivation and professional commitment emerged as recurrent themes, reflecting a general readiness among HCM pediatric staff to engage with innovations aimed at improving patient care. Such human factors are often overlooked in implementation research but can serve as powerful enablers of organizational change, especially in low-resource environments.

Participants expressed optimism that the syndromic antibiogram could optimize empiric antibiotic therapy and reduce inappropriate antimicrobial use. This aligns with evidence from high-income countries, where syndromic antibiograms have been shown to enhance the precision of empiric prescribing and improve patient outcomes [13,36]. Importantly, the Mozambican context highlights that successful implementation depends not only on access to data but also on the alignment of human, technical, and institutional capacities. Although this study was conducted prior to the implementation and roll-out of the syndromic antibiogram, participants’ perspectives suggest that intervention monitoring could be embedded within routine clinical and stewardship activities. This integration would be supported by a structured capacity-building approach, including introductory orientation sessions for clinicians and laboratory staff on the concept, purpose, and interpretation of the syndromic antibiogram. Case-based learning is incorporated into routine clinical meetings and ward rounds, targeted training for laboratory personnel on data entry, validation, and analysis using WHONET, and periodic refresher sessions aligned with syndromic antibiogram updates. Proposed indicators include concordance between empirical prescriptions and syndromic antibiogram recommendations, reductions in inappropriate broad-spectrum antibiotic use, and earlier initiation of appropriate therapy. Review of these indicators through existing ward rounds and antibiotic stewardship committee meetings would support audit-and-feedback and iterative improvement.

Sustainability was perceived to depend primarily on institutionalization rather than continued external funding. Participants emphasized the need to transition from short-term financial incentives towards non-financial motivators, including professional recognition, performance feedback, and training opportunities. Such approaches have been shown to sustain engagement when stewardship activities are aligned with professional identity and supported by leadership [18,19,23,24].

At the policy level, sustainability may be further strengthened through alignment with national AMR strategies. Embedding syndromic antibiograms within national AMR action plans, laboratory-strengthening initiatives, and surveillance frameworks could enhance institutional ownership, facilitate resource allocation, and support scale-up beyond individual hospitals [2,37].

The findings of this study highlight a dynamic interaction between barriers and facilitators that could shape the implementation and sustainability of the syndromic antibiogram. The barriers identified reflect structural, organizational, and resource-related constraints, while the facilitators underscore existing strengths and supportive conditions that foster adoption, collaboration, and long-term use. Table 3 presents a synthesis of these findings, mapping strategies to the main barrier and facilitator themes identified. Measures listed to overcome barriers emphasize the need to strengthen institutional support, enhance resource availability, improve coordination among stakeholders, and establish mechanisms for systematic monitoring. In contrast, strategies that build upon facilitators focus on sustaining motivation, reinforcing teamwork, providing continuous technical and managerial support, and institutionalizing practices that ensure continuity.

Finally, it is important to acknowledge that this study was conducted in a single quaternary hospital in the capital city of Mozambique, which may limit the generalizability of our findings to other healthcare institutions with different organizational structures, resource levels, or institutional cultures. In addition, the study did not include pharmacists among our interviewed participants, despite their central role in antimicrobial stewardship. This omission may limit insights into medication-related governance and supply chain dynamics. Future research should therefore include pharmacists and other relevant healthcare professionals to provide a more comprehensive understanding of the diverse experiences and perspectives related to the implementation of syndromic antibiograms.

This study also has notable strengths. It provides an in-depth, contextually grounded exploration of the anticipated barriers and facilitators at HCM from frontline healthcare providers who are most directly involved in pediatric antibiotic prescribing and diagnostic workflows. This study provides valuable insights from a low-resource setting, where evidence on this topic is limited. It informs the adaptation of implementation frameworks, such as the DAP model, to guide the uptake of innovations in similar contexts. Furthermore, the qualitative depth of the data offers a nuanced understanding of how clinical realities, workflow constraints, and institutional dynamics may influence the feasibility and acceptability of integrating a syndromic antibiogram into routine practice.

## 4. Materials and Methods

### 4.1. Study Design

This qualitative study was conducted prior to the implementation of our syndromic antibiogram intervention to inform the development of effective strategies for its future adoption. It is part of a broader mixed-methods study employing an exploratory sequential design and has been published elsewhere [10].

The study was guided by the Dynamic Adaptation Process (DAP) framework, an implementation science model consisting of four phases: (i) Exploration, (ii) Preparation, (iii) Implementation, and (iv) Sustainability. The DAP framework emphasizes the iterative adaptation of evidence-based practices and accounts for local context, system readiness, and stakeholder engagement [17]. We conducted qualitative interviews with pediatric and laboratory staff at HCM to explore perceived barriers and facilitators to implementing syndromic antibiograms.

### 4.2. Setting

The study was conducted at HCM, the largest quaternary referral hospital in Mozambique. HCM plays a central role in pediatric inpatient care in Mozambique and is representative of other healthcare institutions in LMICs that face significant challenges in microbiological diagnostics, resulting in widespread empiric antibiotic use by its prescribing clinicians (10).

### 4.3. Participants

A purposive sampling strategy was employed to recruit a diverse group of healthcare professionals directly involved in pediatric antibiotic prescribing, laboratory diagnostics, and/or infection control management. Participants included: pediatricians, general physicians, laboratory technicians, and hospital administrators, ensuring representation across key clinical and operational roles relevant to the implementation of the syndromic antibiogram intervention. Inclusion criteria required participants to have at least (1) a minimum of six months of experience in their current role and (2) to be directly involved in antibiotic-related decision-making amongst pediatric patients hospitalized at HCM. Approximately 18 participants were recruited, with the final sample size determined by thematic saturation.

### 4.4. Data Collection

Data were gathered through semi-structured interviews using an interview guide developed in alignment with the DAP framework. The interview guide domains explored a variety of different themes, including current empiric antibiotic prescribing practices; healthcare worker awareness and perceptions about the use of syndromic antibiograms; interdepartmental communication practices that could impact clinical decision making around antibiotic utilization; and finally, the perceived systemic barriers and facilitators that will likely play a role in the implementation and roll-out of the syndromic antibiogram intervention at HCM. All interviews were conducted in Portuguese by trained interviewers, audio-recorded with participant consent, and transcribed verbatim. Field notes were taken to contextualize and enrich the interpretation of participants’ responses. To ensure consistency and accuracy, a senior team member performed quality control by reviewing the original audio recordings against the transcriptions and resolving any discrepancies. The Portuguese transcripts were then translated into English by a bilingual research team member with training and experience in health-related qualitative studies. Translation aimed to maintain both semantic accuracy and cultural meaning. A back-translation process was conducted for a subset of transcripts to verify accuracy and preserve the original meaning by the principal investigator. Any inconsistencies or ambiguities identified during this process were reviewed and discussed among the research team and resolved through consensus to ensure alignment with participants’ intended meanings and to preserve contextual nuance. A data matrix was developed to support systematic organization and comparison of responses, with rows representing participant responses and columns corresponding to the interview questions.

### 4.5. Data Analysis

Data were analyzed using a reflexive thematic approach grounded in a hybrid deductive-inductive coding strategy. Two researchers independently coded each transcript line by line using Microsoft Word and Excel v.2021. A preliminary deductive coding structure was developed based on the core domains of the DAP framework. As the analysis progressed, this structure was iteratively refined to incorporate inductive codes that emerged directly from the data. The coding team met regularly to compare interpretations, resolve discrepancies through consensus, and ensure analytic consistency. When disagreements persisted, a third analyst was consulted.

Participants were purposively sampled, with recruitment guided by thematic saturation, consistent with the DAP framework’s focus on functional roles in the implementation process. Although professional groups were unevenly represented, physicians comprised the largest group, followed by nurses and laboratory technicians. Thematic saturation was assessed iteratively within each professional group. Analysis indicated that no new analytically relevant codes or themes emerged in the final interviews within any group, including laboratory technicians, indicating adequate saturation across functional domains.

Once the coding structure was finalized, codes were organized into categories reflecting key implementation barriers and facilitators, which were subsequently synthesized into overarching themes. To avoid restricting interpretation too early, themes were mapped onto the four phases of the DAP framework only after thematic development was complete: (1) *Exploration*–perceived needs and institutional readiness; (2) *Preparation*–resource availability, training needs, and stakeholder roles; (3) *Implementation*–coordination processes, workflow integration, and structural or technical challenges; and (4) *Sustainability*–long-term feasibility, leadership engagement, and alignment with hospital policies. An audit trail documenting coding decisions, codebook revisions, and analytical memos was maintained throughout the process to strengthen rigor, transparency, and reproducibility.

### 4.6. Ethical Approval

The study was conducted in accordance with the principles of the Declaration of Helsinki and approved by the National Committee for Bioethics in Health (*Comité Nacional de Bioética Para Saúde*, CNBS) (47/CNBS/2023 10 October 2023). Administrative approval was also obtained from the participating hospital (Ref: n^o^ 48.024.1/DCP/HCM/23). To ensure the confidentiality and privacy of the collected data, all participants were assigned unique identifiers, and any personal or institutional information that could potentially identify them was removed from transcripts and reports to maintain anonymity.

## 5. Conclusions

This study shows that, despite persistent systemic and organizational barriers, strong facilitators create a viable pathway for the successful implementation and sustainment of the syndromic antibiogram at HCM. The findings suggest that challenges to antimicrobial stewardship in resource-limited settings arise less from technical limitations than from fragile health systems and uneven institutional support. The Mozambican experience highlights that diagnostic and stewardship innovations like the syndromic antibiogram can both optimize antimicrobial use and reveal structural weaknesses that must be addressed to achieve lasting impact. Sustainable implementation will require coordinated efforts to strengthen leadership, enhance laboratory and data systems, and foster interprofessional collaboration.

## Figures and Tables

**Table 1:** Sociodemographic characteristics of participants.

Participant Characteristics	N = 18 (%)
Sex	
Male	5 (27.8%)
Female	13 (72.2%)
Age-range (years)	
30–35	4 (22.2%)
36–41	5 (27.8%)
42–47	4 (22.2%)
≥48	5 (27.8%)
Occupation	
Pediatrician	5 (27.8%)
General physician	5 (27.8%)
Nurse	5 (27.8%)
Laboratory Technician	3 (16.7%)
Work experience (years)	
6–9	8 (44.4%)
10–13	1 (5.6%)
14–17	4 (22.2%)
≥18	5 (27.8%)

**Table 3:** Strategies mapped to the main barrier and facilitator themes of implementation.

Barriers-Themes	Strategies to Overcome Barriers	Facilitators-Themes	Strategies to Strengthen Facilitators
Weak management support	Advocate for leadership engagement; present evidence on the benefits of SA for patient care	Use of influential champions	Identify and empower clinical champions to promote adoption
Limited baseline knowledge of syndromic antibiograms	Embed ongoing training into routine hospital education through introductory sessions, case-based learning, targeted laboratory training, and periodic refreshers linked to antibiogram update	Recognition of clinical benefits	Share case studies and data demonstrating improved outcomes
Preference for traditional antibiogram	Conduct awareness sessions comparing traditional vs. syndromic methods	Familiarity with traditional antibiogram	Use as a bridge in training to introduce SA concepts
Insufficient clinical samples	Develop standard protocols for adequate sample collection	Team collaboration	Organize joint training and interdisciplinary workshops
Resistance to change	Implement change management strategies; peer-to-peer mentoring	Good acceptance by the clinical team	Reinforce positive feedback and highlight early successes
Lack of laboratory supplies and consumables	Strengthen supply chain planning and procurement where feasible; implement contingency strategies by prioritizing a core set of high-yield syndromes and pathogens and timing antibiogram updates to periods of reagent availability	Technical support among colleagues	Encourage peer learning and establish mentorship systems
Poor communication between the lab and clinicians	Establish clear communication channels; regular joint meetings	Preference for physical/visual formats	Provide user-friendly, visual reports and quick reference tools
Delays in receiving final results	Streamline lab processes; integrate digital reporting tools	Faster results improve prognosis	Continue optimizing turnaround times and highlight patient impact
Lack of time and human resources	Advocate for adequate staffing; reallocate tasks where possible	Teamwork and peer support	Institutionalize team-based approaches and recognize collaborative efforts
Limited institutional support	Integrate SA into hospital policies and quality improvement plans	Monitoring and periodic review	Establish regular audits and feedback systems
Weak monitoring and follow-up	Monitor use through peer review during ward rounds, periodic audits of antibiogram-concordant prescribing, and feedback via stewardship meetings	Maintenance of services/laboratories	Secure long-term funding and technical support
Risk of demotivation if incentives are not sustained	Transition to non-financial incentives (recognition, feedback, training opportunities) and integrate their use into routine clinical roles	Incentives as motivation	Ensure sustainable, fair, and transparent incentive mechanisms

## Data Availability

The qualitative interview transcripts are not publicly available due to ethical restrictions and confidentiality commitments made to the study participants. However, access to the transcripts may be considered upon a formally justified request, subject to evaluation by the responsible researchers and approval by the appropriate ethics committees, ensuring the protection of participants’ identities and full compliance with ethical research principles.
